# Mother phubbing and adolescents’ problematic SNS use: the mediating role of perceived burdensomeness and the moderating role of need to belong

**DOI:** 10.3389/fpsyg.2023.1098707

**Published:** 2023-06-09

**Authors:** Pengcheng Wang, Mingkun Ouyang, Yulong Yin, Biao Li

**Affiliations:** ^1^Shanghai Jiao Tong University, Shanghai, China; ^2^School of Education Science, Guangxi University for Nationalities, Nanning, Guangxi, China; ^3^School of Psychology, Northwest Normal University, Lanzhou, Gansu, China; ^4^School of Journalism and Communication, Faculty of Social Sciences, Renmin University of China, Beijing, China

**Keywords:** mother phubbing, perceived burdensomeness, problematic SNS use, need to belong, adolescents

## Abstract

There is a rapidly growing academic interest on parental phubbing, however, little research has explored the association between mother phubbing and adolescent problematic social networking sites use (PSNSU), the underlying mediating and moderating effects in this relationship are also in need to be uncovered. The present study examined whether mother phubbing would be positively related to adolescent PSNSU, whether perceived burdensomeness would mediate this relationship, and whether need to belong would moderate the associations between mother phubbing and adolescent PSNSU. The hypothesized research model was examined among 3,915 Chinese adolescents (47% of them were boys, mean age = 16.42 ± 0.77 years). The results showed that mother phubbing was positively associated with adolescent PSNSU and perceived burdensomeness mediated this association. Furthermore, need to belong moderated the relationship between perceived burdensomeness and PSNSU, the relationship between mother phubbing and perceived burdensomeness, and the relationship between mother phubbing and PSNSU.

## 1. Introduction

Mobile phones are becoming an essential part of people’s daily life. For example, there are 897 million mobile phone users in China (China Internet Network Information Center, 2020). Smartphones can help satisfy all kinds of need for people, such as information acquisition, interpersonal communication, recreation, and shopping (Nie et al., 2020). However, like the two sides of a coin, mobile phones can also lead to adverse social consequences. With the widespread of mobile phones, researchers are increasingly paying attention to the negative psychological impacts of phubbing. Phubbing is a portmanteau of the words “phone” and “snubbing,” which indicates a scene that an individual is using his/her phone instead of interacting with the people around him/her (Karadag et al., 2015; Chotpitayasunondh and Douglas, 2016, 2018; Roberts and David, 2016; Fang et al., 2020). Phubbing has become a common social phenomenon, for example, a survey indicates that although people think phubbing is both disrespectful and annoying (Aagaard, 2019), 89% of them exhibited phubbing (Rainie and Zickuhr, 2015).

Existing literature reveals that phubbing can lead to a wide range of negative outcomes. For instance, boss phubbing can impair employee performance (Roberts et al., 2017; Roberts and David, 2020; Yasin et al., 2020). Partner phubbing can increase partner depression and impair partner relationship (Roberts and David, 2016; Wang X. et al., 2017; Beukeboom and Pollmann, 2021; Broening and Wartberg, 2022). Moreover, parent phubbing can impair parent–child interaction and relationship (Liu et al., 2019; Wang et al., 2022a,b), increase adolescent depression and loneliness (Xie and Xie, 2020; Bai et al., 2020b; Wang et al., 2021; Li et al., 2022), lead to problematic smartphone use (Hong et al., 2019; Xie et al., 2019; Wang et al., 2020a; Geng et al., 2021), and contribute to cyberbullying perpetration (Qu et al., 2020; Wang et al., 2020b).

Adolescents are heavy users of social networking sites, a growing body of research has focused on adolescents’ problematic SNS use (PSNSU) (e.g., Wang et al., 2018). During adolescence, individuals begin the separation process from their family and peers take on an increasing value. Neverthelss, previous studies find that parent–child interaction, such as father phubbing, is significantly associated with adolescent PSNSU (Wang et al., 2022a). However, little research has examined the association between mother phubbing andadolescent PSNSU. Internet addiction has received continuous attention from researchers due to its adverse influences on people (Diotaiuti et al., 2022a,b). PSNSU (also called SNS addiction, or SNS overuse) can be regarded as a subtype of internet addiction (Wang et al., 2018), it is associated with a series of negative outcomes, such as depression, loneliness, and anxiety (Wang et al., 2018; Yurdagül et al., 2019).

Given the widespread of phubbing in modern society, and given the adverse consequences of PSNSU, it is imperative to examine whether mother phubbing can be related to adolescent PSNSU. Besides, uncovering the mechanisms in this relationship can provide people with a more comprehensive understanding of how mother phubbing can be related to adolescent PSNSU, which would be of great help in shading light on identifying, preventing, and reducing adolescent PSNSU. Based on the above literature, our study recruited a large sample of Chinese adolescents to test (a) whether mother phubbing would be positively associated with PSNSU, (b) whether perceived burdensomeness would be a mediator in the link between mother phubbing and PSNSU, and (c) whether need to belong would moderate the associations between mother phubbing and PSNSU. Moreover, given that mother’s and father’s parenting behaviors may have diverse influences on their children’s development, we choose to focus on mother phubbing in the current study.

### 1.1. Mother phubbing and adolescents’ PSNSU

Mother phubbing can be defined as a scene that a mother is paying attention to her mobile phone instead of interacting with her children around (Bai et al., 2020a). Mother phubbing may lead to adolescent PSNSU. Based on social learning theory (Bandura and Walters, 1977), parents are vital role models for their children, children’s behaviors are often learned by observing their parents. Thus, if a mother often uses mobile phones in front of their children, it is likely that her children will mimic this behavior and become problematic mobile phone users. Given that SNSs are one of the most popluar mobile phone applications among adolescents, and given that PSNSU is closely associated with problematic smartphone use (Karadag et al., 2015). It is logic to posit that mother phubbing can contribute to adolescents’ PSNSU. Existing empirical findings roughly support this assumption. For example, a few cross-sectional studies indicate that parent phubbing is significantly related to adverse outcomes among adolescents, including depression, academic burnout, and problematic smartphone use (Bai et al., 2020a,b; Wang et al., 2020a; Xie and Xie, 2020). Furthermore, a longitudinal study finds that parent phubbing can positively predict adolescents’ problematic smartphone use (Hong et al., 2019). Based on the theory and empirical research reviewed above, it is reasonable to deduce that mother phubbing can be positively related to adolescents’ PSNSU. Nevertheless, little existing research has examined this effect. Thus, we put forward the first hypothesis:

*H1*: Mother phubbing would be positively related to adolescents’ PSNSU.

### 1.2. The mediating role of perceived burdensomeness

Mother phubbing can contribute to adolescents’ perceived burdensomeness. Perceived burdensomeness describes a feeling that other people will be better off if one is gone (Van Orden et al., 2012). As the displacement theory suggests (Kraut et al., 1998; Valkenburg and Peter, 2007), people’s engagement in media will replace their time that could have been spent on offline social interactions. Based on this theory, mother phubbing will reduce a mother’s interaction with her children, which will make the children feel that their mother’s mobile phone is more important than them and their mother does not care about them (Wang et al., 2020a). Thus, it is possible that mother phubbing will make children feel that they are unimportant or even burdens to their mothers. In line with this idea, empirical research indicates that adverse family and interpersonal factors can engender perceived burdensomeness (Van Orden et al., 2012). For instance, family discord positively predicts perceived burdensomeness (Duberstein et al., 2004), social support from family and peer negatively predicts perceived burdensomeness (Hollingsworth et al., 2018; McClay et al., 2020), and interpersonal trust is negatively related to perceived burdensomeness (Hill et al., 2019). However, no existing research ever examined the association between mother phubbing and adolescents’ perceived burdensomeness.

Perceived burdensomeness can lead to PSNSU. As the compensatory internet use theory suggests, people often use the internet to escape their real life problems or alleviate their negative moods, which can lead to problematic internet use (Kardefelt-Winther, 2014). Given that perceived burdensomeness indicates an unmet social need in the real world (Van Orden et al., 2012; Chu et al., 2018), it is possible people high in perceived burdensomeness may turn to the online social world to compensate for it, which makes them more likely to become problematic social networking sites users. Besides, based on the compensatory internet use theory, people with negative psychological conditions are more likely to become problematic internet user (Kardefelt-Winther, 2014). Given that perceived burdensomeness represents a negative mental status, which is associated with a wide range of adverse outcomes such as anxiety (Hill et al., 2018), depression (Carrera and Wei, 2017), problematic alcohol use (Gallyer et al., 2018), posttraumatic stress symptoms (Short et al., 2020), and even suicide attempt (Ma et al., 2016; Buitron et al., 2017; Mitchell et al., 2018; Hains et al., 2019), it is logically possible that perceived burdensomeness will lead to PSNSU. Nevertheless, little research has explored the association between perceived burdensomeness and PSNSU. Based on the above literature, the following hypotheses were established:

*H2*: Mother phubbing would be positively associated with perceived burdensomeness.

*H3*: Perceived burdensomeness would be positively associated with adolescents’ PSNSU.

*H4*: Perceived burdensomeness would mediate the association between mother phubbing and adolescents’ PSNSU.

### 1.3. The moderating role of need to belong

Although mother phubbing may contribute to adolescents’ PSNSU, it is not likely that all adolescents would be equally influenced by it. Existing literature roughly supports this view. For instance, while a few studies found that partner phubbing was negatively associated with relationship satisfaction (Roberts and David, 2016; Wang X. et al., 2017), another study showed that this effect was not significant (Wang et al., 2019). Besides, prior studies also indicated that the effect of parental phubbing on adolescent outcomes can be moderated by certain personal traits (Bai et al., 2020a,b; Wang et al., 2020a). Thus, it is necessary to examine whether the association between mother phubbing and adolescents’ PSNSU would be moderated by certain personal traits. In this study, we examined the assumption that need to belong would moderate the relationships between mother phubbing and adolescents’ PSNSU.

Need to belong describes the extent to which a person desires to establish and maintain social bonds with other people (Baumeister and Leary, 1995). As the need to belong hypothesis suggests, need to belong is as an extremely persuasive, powerful, and fundamental motivation, which is associated with a wide range of cognitional, emotional, and behavioral outcomes (Baumeister and Leary, 1995). People with a high level of need to belong often have a high demand for interpersonal bonds, they are more likely to participate in activities that promote their social relationships. Given that the primary function of social networking sites is helping people to connect with each other, it is possible that people high in need to belong will have a high level of PSNSU. Prior research supports this assumption by showing that need to belong is positively related to PSNSU (Yin et al., 2019).

Moreover, need to belong may moderate the associations between mother phubbing and adolescents’ PSNSU. As the differential-susceptible hypothesis indicates (Belsky and Pluess, 2009), people with diverse personal traits may response to the same environmental differently. According to existing literature, a high level of need to belong often associates with a series of negative consequences, such as borderline personality disorder and attachment anxiety (Sato et al., 2020). Therefore, a high level of need to belong can be regarded as a negative personal trait. Given that mother phubbing is a negative environmental factor for adolescent development (Bai et al., 2020a), it is logic to posit that need to belong may moderate the associations between mother phubbing and adolescents’ PSNSU. To our knowledge, no research has tested the moderating effect of need to belong in these associations. Based on the above literature, we establish the following hypothesis:

*H5*: Need to belong would moderate the associations between mother phubbing and adolescents’ PSNSU.

### 1.4. The present study

**Figure 1:**
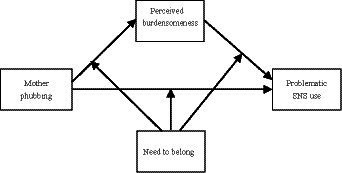
The assumed moderated mediation model.

Collectively, the main aims of our study were as follows. Firstly, our study examined whether mother phubbing would be positively associated with adolescent PSNSU. Secondly, our study tested whether perceived burdensomeness would mediate the association between mother phubbing and adolescent PSNU. At last, our study examined whether need to belong would moderate the associations between mother phubbing and adolescents’ PSNSU (see Figure 1).

## 2. Methods

### 2.1. Participants

The convenience sampling method was used to collect data in this study. Three thousand nine hundred and fifteen students participated in the present study, they were recruited from 4 senior high schools from Jiangsu, Jiangxi, Shandong, and Hebei, China. Before the survey, the informed consent was acquired from the students and their teachers. Regarding their demographic information, 47% of them were boys, the mean age of them was 16.42(±0.77) years, 78% of them were from the rural areas, and 21% of them were the only-child in their family. Besides, 11% of the participants’ father and 10% of the participants’ mother had a high school degree or above. The study variables involved in this study were part of our survey studies, including demographic information, mother phubbing, PSNSU, perceived burdensomeness, and need to belong.

### 2.2. Measures

#### 2.2.1. Mother phubbing

Mother phubbing was measured by the Mother Phubbing Scale (Bai et al., 2020a), which was modified from the Generic Scale of Being Phubbed (Chotpitayasunondh and Douglas, 2018). This scale consists of 22 items (e.g., “I tell my mother that she interacts with her phones too much”). Participants rated each item on a 7-point Likert scale from 1 (strongly disagree) to 7 (strongly agree), with higher averaged scores representing higher levels of mother phubbing. This scale showed great reliability in the present study (Cronbach’s α = 0.91).

#### 2.2.2. PSNSU

PSNSU was measured by the Chinese version of the Social Networking Sites Addiction Scale (Wang et al., 2018; Yin et al., 2019), which was modified from the Facebook Intrusion Questionnaire (Elphinston and Noller, 2011). This scale consists of 8 items (e.g., “I lose track of how much I am using social networking sites”). Participants rated each item on a 7-point Likert scale from 1 (never) to 7 (always), with higher averaged scores representing higher levels of PSNSU. This scale showed good reliability in the present study (Cronbach’s α = 0.87).

#### 2.2.3. Perceived burdensomeness

Perceived burdensomeness was measured by the Perceived Burdensomeness subscale of the Interpersonal Needs Questionnaire (Van Orden et al., 2012). This scale consists of 6 items (e.g., “These days the people in my life would be happier without me”). Participants rated each item on a 7-point Likert scale from 1 (strongly disagree) to 7 (strongly agree), with higher averaged scores representing higher levels of perceived burdensomeness. This scale showed great reliability in the present study (Cronbach’s *α* = 0.93).

#### 2.2.4. Need to belong

Need to belong was measured by the Chinese version of the Single Item Need to Belong Scale (Nichols and Webster, 2013; Wang P. et al., 2017; Yin et al., 2019), which has been proven to have good reliability and validity. The item included in the scale was “I have a strong need to belong.” Participants rated this item on a 7-point Likert scale from 1 (strongly disagree) to 7 (strongly agree), with higher scores representing higher levels of need to belong.

### 2.3. Procedure

The research was conducted in the participants’ classrooms in June 2019. The survey was presented to the participants in paper questionnaires. The investigators were trained to ensure that the data acquisition process was standardized before the survey. The students were notified that their participations were voluntary, and they were free to withdraw from the survey if they wanted to. Each participant received a small gift when finishing all the measurements.

### 2.4. Statistical analyses

Firstly, the samples contained missing data were not included in the statistics, at last, 3915 samples were included in the analyses. Second, we analyzed the descriptive statistics and Pearson-correlations with IBM SPSS 26.0. Third, we used the PROCESS 3.0 macro Model 4 to examine the mediating effect of perceived burdensomeness (Hayes, 2017). At last, we applied the PROCESS 3.0 macro Model 59 to examine the moderated mediation model (Hayes, 2017). In the present study, mother phubbing was set as *X*, problematic SNS use was set as *Y*, perceived burdensomeness was set as *M*, and need to belong was set as W in the PROCESS 3.0 macro (Hayes, 2017). In the mediation and moderated mediation analyses, participants’ gender, age, birth place, sibling situation, and their father and mother’s education backgrounds were included as covariates. In the moderated mediation analyses, all variables included were standardized before calculation. Moreover, we used the bootstrap confidence intervals (CIs) to verify whether the effects in Model 4 and Model 59 were significant drawing on 5,000 random samples, an effect would be regarded as significant when the CIs do not contain zero.

## 3. Results

### 3.1. Preliminary analyses

The descriptive statistic and Pearson-correlations for the variables are presented in Table 1. Mother phubbing was positively related to adolescents’ PSNSU, perceived burdensomeness, and need to belong, respectively. Perceived burdensomeness was positively related to PSNSU, and need to belong, respectively. PSNSU was positively related to need to belong.

**Table 1:** Descriptive statistics and correlations between variables.

	*M*	*SD*	1	2	3	4	5	6	7	8	9
1. Gender	0.47	0.50	1								
2. Age	16.42	0.77	−0.12**	1							
3. District	0.78	0.42	0.02	0.11**	1						
4. Sibling	0.79	0.44	0.14***	−0.05**	0.28***	1					
5. FEDU	2.37	1.02	−0.03	−0.09**	−0.35***	−0.23***	1				
6. MEDU	2.24	1.04	0.002	−0.10***	−0.37***	−0.24***	0.61***	1			
7. MPhubbing	2.25	0.92	−0.02	−0.02	−0.06**	−0.02	0.02	0.05**	1		
8. PB	2.29	1.46	−0.09***	−0.04**	−0.001	−0.02	−0.004	−0.01	0.25***	1	
9. PSNSU	2.97	1.31	−0.03	0.001	−0.03	0.004	0.02	0.03	0.25***	0.38***	1
10. NTB	3.79	1.86	0.04*	−0.07***	−0.07***	0.03	0.02	0.02	0.11***	0.14***	0.19***

*N* = 3,915. For gender, 1 = male, 0 = female. For district, 1 = rural, 0 = urban. For sibling, 1 = has one or more siblings, 0 = the only-child in family. FEDU, father’s education level; MEDU, mother’s education level; MPhubbing, mother phubbing; PB, perceived burdensomeness; PSNSU, problematic SNS use; NTB, need to belong. *p < 0.05, **p < 0.01, and ***p < 0.001.

### 3.2. Testing for mediation effect

The present study hypothesized that perceived burdensomeness would mediate the association between mother phubbing and adolescents’ PSNSU, which was tested with Model 4 of the PROCESS 3.0 macro (Hayes, 2017). As shown in Table 2, mother phubbing was positively related to perceived burdensomeness (*b* = 0.40, *p* < 0.001), which in turn was positively related to adolescents’ PSNSU (*b* = 0.30, *p* < 0.001). At the same time, mother phubbing was positively associated with adolescents’ PSNSU (*b* = 0.23, *p* < 0.001). Thus, perceived burdensomeness partially mediated the association between mother phubbing and adolescents’ PSNSU (indirect effect = 0.12, se = 0.01, CI = 0.10–0.14). This mediation effect accounted for 34% of the total effect.

**Table 2:** Testing the mediation effect of perceived burdensomeness.

	Model 1 (PB)				Model 2 (PSNSU)			
Predictors	Coefficient	SE	LLCI	ULCI	Coefficient	SE	LLCI	ULCI
Gender	−0.25***	0.05	−0.33	−0.16	0.01	0.04	−0.06	−0.09
Age	0.07*	0.03	0.01	0.13	−0.01	0.03	−0.06	0.04
District	0.02	0.06	−0.10	0.14	−0.06	0.05	−0.16	0.04
Sibling	0.002	0.05	−0.11	0.11	0.07	0.05	−0.02	0.16
FEDU	0.01	0.03	−0.05	0.06	0.01	0.02	−0.04	0.06
MEDU	−0.02	0.03	−0.08	0.06	0.03	0.02	−0.02	0.07
MPhubbing	0.40***	0.02	0.35	0.44	0.23***	0.02	0.19	0.27
PB					0.30***	0.01	0.28	0.33
*R^2^*	0.07				0.17			
*F*	43.13***				100.47***			

N = 3,915. MPhubbing, mother phubbing; PB, perceived burdensomeness; PSNSU, problematic SNS use. *p < 0.001; ***p < 0.001.

### 3.3. Testing for moderated mediation effect

**Figure 2:**
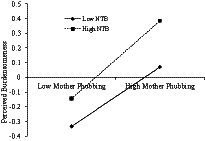
Need to belong (NTB) moderates the association between mother phubbing and perceived burdensomeness (*N* = 3,915).

**Figure 3:**
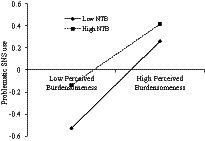
Need to belong (NTB) moderates the association between perceived burdensomeness and problematic SNS use (*N* = 3,915).

**Figure 4:**
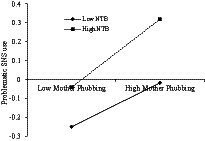
Need to belong (NTB) moderates the association between mother phubbing and problematic SNS use (*N* = 3,915).

The present study assumed that need to belong would moderate the associations between mother phubbing and adolescents’ PSNSU, which was examined with Model 59 of the PROCESS 3.0 macro (Hayes, 2017). As presented in Table 3, the results indicated that need to belong moderated the link between mother phubbing and perceived burdensomeness (*β* = 0.031, *p* < 0.05). To make this finding visual, we plotted the effect of mother phubbing on perceived burdensomeness at a high and a low level of need to belong (meant ± 1 SD, respectively; see Figure 2). Simple slope analyses showed that the association between mother phubbing and perceived burdensomeness was significantly stronger for adolescents with a high level of need to belong (*β_simple_* = 0.27, *t* = 12.94, *p* < 0.001) compared to adolescents with a low level of need to belong (*β_simple_* = 0.20, *t* = 9.03, *p* < 0.001). Besides, need to belong moderated the link between perceived burdensomeness and adolescents’ PSNSU (*β* = −0.058, *p* < 0.001). To make this finding visual, we plotted the effect of perceived burdensomeness on PSNSU at a high and a low level of need to belong (meant ± 1 SD, respectively; see Figure 3). Simple slope analyses showed that the association between perceived burdensomeness and PSNSU was significantly stronger for adolescents with a low level of need to belong (*β_simple_* = 0.39, *t* = 16.94, *p* < 0.001) compared to adolescents with a high level of need to belong (*β_simple_* = 0.28, *t* = 15.21, *p* < 0.001). Moreover, need to belong moderated the association between mother phubbing and PSNSU (*β* = 0.032, *p* < 0.05). To make this finding visual, we also plotted the effect of mother phubbing on PSNSU at a high and a low level of need to belong (meant ± 1 SD, respectively; see Figure 4). Simple slope analyses showed that the association between mother phubbing and PSNSU was significantly stronger for adolescents with a high level of need to belong (*β_simple_* = 0.18, *t* = 9.06, *p* < 0.001) compared to adolescents with a low level of need to belong (*β_simple_* = 0.12, *t* = 5.35, *p* < 0.001).

**Table 3:** Testing the moderating effect of need to belong.

	Model 1 (PB)		Model 2 (PSNSU)	
Predictors	*β*	*t*	*β*	*t*
Gender	−0.09	−5.7***	0.001	0.05
Age	0.04	2.81**	0.004	0.30
District	0.02	0.38	−0.01	−0.50
Sibling	−0.01	−0.37	0.02	1.02
FEDU	0.001	0.07	0.01	0.39
MEDU	−0.01	−0.64	0.02	1.12
MPhubbing	0.23	15.11***	0.15	9.87***
NTB	0.13	8.16***	0.14	9.30***
MPhubbing × NTB	0.031	2.10*	0.032	2.22*
PB			0.34	21.61***
PB × NTB			−0.058	−4.16***
*R^2^*	0.09		0.19	
*F*	42.04***		83.75***	

*N* = 3,915. MPhubbing, mother phubbing; PB, perceived burdensomeness; PSNSU, problematic SNS use; NTB, need to belong. *p < 0.05, **p < 0.01, and ***p < 0.001.

## 4. Discussion

Although the research attention on parental phubbing is growing rapidly in the past few years (Bai et al., 2020b; Wang et al., 2020a; Xie and Xie, 2020), little research has reported the association mother phubbing and adolescents’ PSNSU, the underlying mediating and moderating effects in this relationship are also in need to be explored. Our study tested whether perceived burdensomeness would be a mediator in the association between mother phubbing and adolescents’ PSNSU, and whether need to belong would be a moderator in the associations between mother phubbing and adolescents’ PSNSU. After controlling for the covariates, the findings indicated that mother phubbing significantly and positively related to adolescents’ PSNSU, perceived burdensomeness was a mediator in the link between mother phubbing and adolescents’ PSNSU. Moreover, need to belong was a moderator in the association between mother phubbing and adolescents’ PSNSU, in the association between mother phubbing and perceived burdensomeness, and in the association between perceived burdensomeness and adolescents’ PSNSU. All these findings are discussed in the following parts.

### 4.1. Mother phubbing and adolescents’ PSNSU

Prior research indicates that parental phubbing has a series of adverse influences on child/adolescent development, such as increased depression and problematic smartphone use, and impaired self-esteem (Hong et al., 2019; Liu et al., 2019; Bai et al., 2020b). However, little research has examined the unique effect of mother phubbing on adolescent PSNSU. Our study goes beyond existing literature by indicating mother phubbing is significantly and positively related to adolescents’ PSNSU. As we are aware, our study is the first to report this finding. This finding is in line with the social learning theory (Bandura and Walters, 1977), which argues that parents are one of the main role models for their children. Thus, if mother phubbing takes place frequently, adolescents may mimic this behavior and become problematic SNS users.

### 4.2. The mediating role of perceived burdensomeness

Our study indicates that mother phubbing was significantly and positively linked to adolescents’ perceived burdensomeness, which in turn was significantly and positively linked to adolescent PSNSU. Put differently, perceived burdensomeness was a mediator in the association between mother phubbing and adolescents’ PSNSU. Thus, perceived burdensomeness should be regarded one of the explanatory factors for why mother phubbing can lead to adolescents’ PSNSU.

In addition to the mediating effect, the two separate pathways in the mediating model are also worth remarking. The first pathway of the mediating model showed that mother phubbing was significantly and positively related to perceived burdensomeness. To the best of our knowledge, this relationship has not been examined by prior research. This finding supports the displacement hypothesis (Kraut et al., 1998), which suggests that people’s media use will replace their time that could have been spent on interacting with others in the real world. Based on this theory, mother phubbing would inevitably replace mothers’ meaningful interactions with their children. Thus, mother phubbing can reduce and impair mothers’ responses to their children and make their children feel less needed or cared by their mother, which makes their children feel that they are a burden.

The second pathway of the mediating model showed that perceived burdensomeness was significantly and positively related to adolescents’ PSNSU, which also has not been examined by prior studies. This finding is in congruent with the compensatory internet use theory (Kardefelt-Winther, 2014), which suggests that people often turn to the internet to escape from unsatisfied real-life situations or negative psychological status. Given that perceived burdensomeness represents a form of unmet social bonds, which is closely related to a range of negative psychological symptoms (Van Orden et al., 2012; Buitron et al., 2017; Vanyukov et al., 2017; Chu et al., 2018; Gallyer et al., 2018), it is logical to find that perceived burdensomeness was positively related to PSNSU.

### 4.3. The moderating role of need to belong

Our study also explored whether need to belong, as a fundamental personal trait, would be a moderator the associations between mother phubbing and adolescents’ PSNSU. The results showed that need to belong moderated all the three pathways in the research model. Each of these findings are discussed as follows.

Firstly, need to belong exacerbated the association between mother phubbing and perceived burdensomeness. To be specifc, the relationship between mother phubbing and perceiced burdensomeness was significantly stronger among adolescents with a high level of need to belong than adolescents with a low level of need to belong. This finding suopprts the differential-susceptible hypothesis (Belsky and Pluess, 2009), which suggests that negative personal trati can exacerbate the negative influence stimulated by negatvie enviromental factors like mother phubbing.

Secondly, need to belong exacerbated the association between mother phubbing and adolescents’ PSNSU. Specially, the association between mother phubbing and PSNSU was much stronger among adolescents with a high level of need to belong than adolescents with a low level of need to belong. This finding is also in line with the differential-susceptible hypothesis (Belsky and Pluess, 2009), which argues that vulnerable personal trait may exacerbate the adverse effects caused by negative environmental factors. Given that people high in need to belong are more sensitive about their interpersonal relationships, it is reasonable to find that the relationship between mother phubbing and PSNSU was much stronger among them.

Thirdly, need to belong moderated the association between perceived burdensomeness and PSNSU. To be specific, the association between perceived burdensomeness and PSNSU was much stronger for adolescents with a low level of need to belong than for adolescents with a high level of need to belong, which was different from the expectation that need to belong would exacerbate this relationship. One possible reason is that perceived burdensomeness is closely associated with adolescents’ PSNSU. In other words, for adolescents high in perceived burdensomeness, they already have a high level of PSNSU. Thus, there may be a ceiling effect, which makes the link between perceived burdensomeness and PSNSU weaker among adolescents with a high level of need to belong.

### 4.4. Implications

The results in our study have some illuminating theoretical and practical implications. Firstly, our study highlights the significance of mother phubbing on the formation and maintenance of adolescents’ PSNSU. The literature on phubbing is still in its infancy, little research has ever explored the link between parental phubbing and adolescents’ PSNSU, our study provides us with a more comprehensive understanding of the negative consequences caused by phubbing. Our study shows that phubbing in the family context can lead to PSNSU, which reminds mothers to reduce their mobile phone use in front of their children. This finding suggests that if mothers use their phones when they are around their children, it can set a negative model for the children and may lead the children to PSNSU. Thus, mothers should be aware that they are important role models for their children when it comes to digital device use. Secondly, our study shows that perceived burdensomeness is a proximal explanatory factor for why mother phubbing is positively related to adolescents’ PSNSU. Therefore, intervention effects that reduce perceived burdensomeness may be of significant help in reducing adolescents’ PSNSU. Existing research suggests that unmet interpersonal relationships are key factors predicting burdensomeness (Van Orden et al., 2012), thus, it is important to provide adolescents with good interpersonal relaitonships. Last but not least, our study shows that need to belong moderates all the three paths between mother phubbing and adolescents’ PSNSU, which can provide the researchers with a sophisticated understanding of how the dynamics of mother phubbing (as an environmental factor) and need to belong (as a personal trait) can jointly influence adolescents’ PSNSU. It also reminders educators and parents to pay more attention to adolescents with certain traits like need to belong when facing mother phubbing.

### 4.5. Limitations and future directions

Our study establishes a basis for studying the influence of mother phubbing on adolescents’ PSNSU, which provides some novel findings in the literature, However, several aspects of our study are in need to be improved. Firstly, our study only recruited a large sample of adolescents from 4 provinces in China, given that people in the western and eastern countries may response to interpersonal factors differently, it is necessary to check whether the findings obtained in our study can apply to people from other cultural backgrounds. Secondly, our study indicates that mother phubbing is significantly and positively related to adolescents’ PSNSU, it is worthy testing whether mother phubbing can contribute to adolescents’ other problematic media use, for instance, problematic mobile phone games use. Thirdly, our study only collected self-reported data from adolescents in a cross-sectional designed paradigm, future research can collect mother-adolescent paired longitudinal data to further confirm these findings. Fourthly, our study used a one-item need to belong scale, future studies can use the 10-item need to belong scale to replicate our findings (Leary et al., 2013). Last but not least, it is possible that there can be other important mediators and moderators in the association between mother phubbing and adolescent problematic SNS use, it would bebenefical to examine them in future studies.

## 5. Conclusion

Collectively, our study shows that mother phubbing is positively related to adolescents’ PSNSU. Besides, the mediation analyses indicate that perceived burdensomeness is a proximal explanatory factor for how mother phubbing relates to adolescents’ PSNSU. Furthermore, moderated-mediation analyses suggests that need to belong moderates the relationship between mother phubbing and perceived burdensomeness, the relationship between perceived burdensomeness and PSNSU, and the relationship between mother phubbing and PSNSU among adolescents.

## Data availability statement

The raw data supporting the conclusions of this article will be made available by the authors, without undue reservation.

## Ethics statement

The studies involving human participants were reviewed and approved by Renmin University of China. Written informed consent to participate in this study was provided by the participants’ legal guardian/next of kin.

## Author contributions

PW: conceptualization, validation methodology, software, data curation, visualization, writing original draft, and investigation. MO: validation methodology, software, and data curation. YY: software, data curation, and visualization. BL: conceptualization and supervision. All authors contributed to and have approved the final manuscript.

## Funding

This research was supported by National Social Science Foundation of China (No. 20&ZD319).

## Conflict of interest

The authors declare that the research was conducted in the absence of any commercial or financial relationships that could be construed as a potential conflict of interest.

The reviewer DL declared a shared affiliation with the author BL to the handling editor at the time of review.

## Publisher’s note

All claims expressed in this article are solely those of the authors and do not necessarily represent those of their affiliated organizations, or those of the publisher, the editors and the reviewers. Any product that may be evaluated in this article, or claim that may be made by its manufacturer, is not guaranteed or endorsed by the publisher.
